# JNK/AP1 Pathway Regulates MYC Expression and BCR Signaling through Ig Enhancers in Burkitt Lymphoma Cells

**DOI:** 10.7150/jca.34055

**Published:** 2020-01-01

**Authors:** Xiaoling Ding, Xiaoying Wang, Xueting Zhu, Jie Zhang, Yiqing Zhu, Xiaoyi Shao, Xiaorong Zhou

**Affiliations:** 1 Department of Gastroenterology, The Affiliated Hospital of Nantong University, 20 Xisi Road, Nantong, Jiangsu 226001, China.; 2 Department of Immunology, Nantong University, School of Medicine, 19 Qixiu Road, Nantong, Jiangsu 226001, China.

**Keywords:** Lymphoma, MYC, Enhancer, AP1, JNK.

## Abstract

In Burkitt lymphoma (BL), a chromosomal translocation by which the MYC gene is fused to an immunoglobulin (Ig) gene locus is frequently found. The translocated MYC gene is overexpressed, which is the major driver of BL tumorigenesis. Studies have shown that Ig enhancers are essential for MYC overexpression, but the involved mechanisms are not fully understood. In addition, the survival of BL cells relies on B-cell receptor (BCR) signaling, which is determined by the levels of Ig molecules expressed on the cell surface. However, whether MYC has any impact on Ig expression and its functional relevance in BL has not been investigated. Herein, we show that MYC upregulates Ig kappa (Igκ) expression in BL cells through two Igκ enhancers, the intronic enhancer (Ei) and the 3ʹ enhancer (E3ʹ). Mechanistically, by activating the JNK pathway, MYC induces the phosphorylation of c-Fos/c-Jun and their recruitment to AP1 binding sites in the Igκ enhancers, leading to the activation of the enhancers and subsequent Igκ upregulation. The AP1-mediated activation of the Igκ enhancers is also required for the expression of the translocated MYC gene, indicating positive feedback for the MYC overexpression in BL cells. Importantly, interrupting the JNK pathway inhibits both Igκ and MYC gene expression and suppresses BL cell proliferation. Our study not only reveals a novel mechanism underlying MYC overexpression in BL but also suggests that targeting the JNK pathway may provide a unique strategy to suppress BL tumorigenesis.

## Introduction

Burkitt lymphoma (BL) cells are characterized by a translocation fusing the oncogene MYC gene located on chromosome 8 to one of the immunoglobulin (Ig) gene loci 1, 2. The translocated MYC gene is persistently overexpressed, which plays a major role in the pathogenesis of BL 3. MYC overexpression relies on the presence of nearby Ig enhancers: the 3ʹ enhancer of the Ig heavy chain (IgH) gene in cells with IgH/MYC translocations and the intronic enhancer (Ei) and 3ʹ enhancer (E3ʹ) of the Ig kappa (Igκ) gene in cells with Igκ/MYC translocations 4. Notably, in BL cells, transcription of the translocated MYC gene is initiated preferentially at the promoter P1, probably through interactions between the Ig enhancers and the P1 promoter, whereas in normal cells, transcription of the MYC gene is mainly driven by the P2 promoter 5, 6. Using reporter assays, Nicola et al. showed that deletions of the NFκB and PU.1 binding sites in Ei and E3ʹ, respectively, reduced P1 but not P2 promoter activity, suggesting that the two transcription factors (TFs) are important for MYC dysregulation in BL 7. However, multiple TFs, including activator protein 1 (AP1), YY1, TCF3 (E2A), and STAT5, can bind to Ei and E3ʹ in different contexts 8-11, and their roles in MYC overexpression in BL are not fully understood.

B cell receptor (BCR) is composed of four B cell-specific proteins: the Ig heavy and Ig light chains and the Igα (CD79A) and Igβ (CD79B) polypeptides. Signaling that is transduced through the Igα/Igβ heterodimer governs the development and function of normal B cells. Abnormal BCR signaling is directly involved in the pathogenesis of B cell malignancies 12, 13, and targeting the BCR signaling pathway reduced the cell proliferation of various types of lymphomas, including BL 14-17. Given the concurrent dysregulation of MYC and BCR in BL cells and their functional relevance in BL pathogenesis 18-20, we hypothesized that MYC might upregulate BCR signaling and thus promote BL progression. Here, we show that MYC enhances Igκ expression in BL, which is dependent on JNK activation and the resulting recruitment of AP1 to Igκ enhancers. Moreover, the binding of AP1 to Igκ enhancers is important for the activity of MYC promoter P1, indicating a key role of AP1 in MYC dysregulation in BL cells. Importantly, targeting the JNK pathway diminished AP1 binding to Igκ enhancers and suppressed both MYC and Igκ expression in BL cells. These findings reveal new mechanisms involved in BL tumorigenesis and have implications for targeted therapy of BL.

## Materials and methods

### Cell culture

Raji and DG75 cells (BL cell lines) were purchased from the American Type Culture Collection (ATCC, Manassas, VA) and were cultured in RPMI-1640 (GIBCO, Grand Island, NY) supplemented with 10% fetal bovine serum (FBS). The JNK inhibitor SP600125 and MYC inhibitor 10058-F4 were purchased from Selleckchem (Houston, TX), and both were dissolved in DMSO to make the stock solutions.

### Plasmids and transfection

The SMARTpool of small interfering RNAs for MYC, Igκ and the nontargeting control were purchased from Dharmacon (Lafayette, CO). The MYC-expressing vector was generated by cloning the MYC cDNA into a pcDNA3.1 vector (Invitrogen, Waltham, MA). For transfection, 2×10^6^ cells were resuspended in 100 μl of buffer (Engreen, Beijing, China), and electrotransfection was performed with 2.5 µg of plasmid using a Celetrix Electroporator (Celetrix, LLC. Shanghai, China). The depletion of CD79a was performed using the Lenti-CRISPR-V2 vector (Addgene #52961) to introduce Cas9 and the single guide RNA (sgRNA), as previously described 21, and the sgRNA sequences are provided in the Supplementary Information (Table S1).

### Real-time PCR and PCR

RNA was extracted using the RNeasy Mini Kit (Qiagen, Germantown, MD), and cDNA was synthesized using the SuperScript VILO cDNA Synthesis Kit (Thermo Fisher, Richardson, TX). Real-time quantitative PCR was performed by using the QuantStudio 5 system (Thermo Fisher) and iTaq™ Universal SYBR Green Supermix (Bio-Rad, Hercules, CA). For data analysis, the 2^-ΔΔCT^ method was used to calculate the fold changes. GAPDH expression was considered to be unaffected under our treatment conditions, and GAPDH was used as a reference gene. Each experiment was run in triplicate, and the error bars represent the range of the fold changes calculated from three or four independent experiments. The primer sequences used for real-time PCR are provided in the Supplementary Information (Table S1).

### Western blotting

Western blotting was performed using whole cell lysates. Aliquots of total protein (20-50 μg per lane) were electrophoresed on a 10% SDS-polyacrylamide gradient gel and transferred to nitrocellulose membranes (Millipore, Bedford, MA). The membranes were incubated at 4 °C overnight with anti-GAPDH, MYC, c-Jun, p-c-Jun, c-Fos, p-c-Fos, JNK, p-JNK, or Igκ monoclonal antibody (all purchased from Abcam, Cambridge, MA). After rinsing in buffer wash, the membranes were incubated with a horseradish peroxidase-conjugated secondary antibody (Santa Cruz Biotechnology, Dallas, TX) diluted 1:10,000-30,000, followed by development with enhanced chemiluminescence reagents (Amersham, Little Chalfont, UK).

### Dual luciferase reporter assay

The reporter vectors pGL4-P1, pGL4-P1-E3′, pGL4-P1-Ei-ΔAP1, and pGL4-P1-E3ʹ-ΔAP1 were generated by modifying the pGL4-Basic vector purchased from Promega (Madison, WI); the details regarding vector construction are provided in the Supplemental Information (DOC S1). A luciferase reporter assay was performed with the Dual-Luciferase Reporter Assay System (Promega) according to the manufacturer's instructions. Briefly, cells were transfected with 2.5 µg of DNA in 24-well plates using Lipofectamine 3000 (Thermo Fisher), and the luciferase activity was measured 48 h after transfection using a Junior LB luminometer (Berthold Technologies, Bad Wildbad, Germany). The assays were carried out in duplicate in 3 independent experiments.

### Coimmunoprecipitation assays (Co-IP)

IP lysis buffer (Thermo Fisher) was used to lyse Raji cells, and immunoprecipitation (IP) was performed using the Dynabeads Protein G Immunoprecipitation Kit (Invitrogen) according to the manufacturer's instructions. Briefly, 2 x 10^7^ Raji cells were harvested, and cell lysates were prepared using cold IP lysis buffer containing 1 X Halt™ Protease Inhibitor (Thermo Fisher). Five micrograms of antibody were coated on 1.5 mg of Dynabeads and washed with cold IP lysis buffer three times. The antibody-conjugated beads were incubated with 1.0 mg of protein lysate at 4ºC overnight. Then, the beads were washed extensively, and the IP products were harvested using denaturing elution and subjected to western blot analysis to detect the protein-protein interactions.

### Chromatin immunoprecipitation assays (ChIP)

ChIP analysis was performed as previously described 11. Chromatin solutions were precipitated overnight at 4 °C using c-Jun antibody or rabbit Ig control (Abcam, Cambridge, MA). The input DNA and the immunoprecipitated DNA were extracted using Qiagen spin columns and were analyzed by PCR or real-time PCR assays using Ei and E3′ specific primers (Table S1).

### Cell proliferation and apoptosis assay

A total of 1×10^4^ cells in 200 μl of medium were seeded in each well of 96-well cell culture plates, and the proliferation assay was performed at 6 h, 12 h, 24 h, and 48 h after incubation using a Cell Counting Kit-8 (Dojindo, Kumamoto, Japan). The cell numbers were evaluated by measuring the absorbance at 450 nm with an MR7000 plate reader (Dynatech Laboratories, Chantilly, VA). The apoptosis rate of cells was measured by an Annexin V-PE/7-AAD double staining kit (BioLegend, San Diego, CA) according to the manufacturer's instructions. In brief, 5 × 10^5^ cells were harvested by centrifugation at 2000 rpm for 5-10 min and resuspended in 300 µL binding buffer, followed by a 15 min incubation with 5 μl Annexin V-PE and 5 μl 7-AAD in the dark at 37 °C. Then, flow cytometry analysis was employed to detect both early and late apoptotic cells that were positive for Annexin-V staining.

### Soft agar assay

A soft agar assay was performed in 12-well plates as described previously 22. Briefly, 1.0 ml of agar (bottom layer) was prepared by dissolving 0.5% agarose (Lonza, Basel, Switzerland) in R25 medium (RPMI-1640 supplemented with 25% FBS and 1% antibiotic-antimycotic solution, all obtained from Thermo Fisher). Once the bottom agar layer solidified, 0.5 x 10^4^ cells were resuspended in 1.0 ml of top layer agar (0.37% agarose dissolved in R25 medium) and immediately seeded on top of the bottom agar in triplicate. After the top layer solidified, 1.0 ml of R25 medium was added on top, and the medium was changed every three days. After four weeks, the colonies were fixed in 1 ml of 10% MeOH/10% acetic acid for 10 min and stained with 500 µl of crystal violet (0.005%) for 1-2 h. After staining, the crystal violet stain was removed, and the plate was washed with PBS for 4 h followed by the counting of the colonies.

### Statistical analysis

All statistical analyses were carried out using GraphPad Prism for Windows. The quantitative variables were analyzed by Student's t-test. All statistical analyses were two-sided, and p <0.05 was considered statistically significant.

## Results

### MYC enhances Igκ expression in BL cells

**Figure 1:**
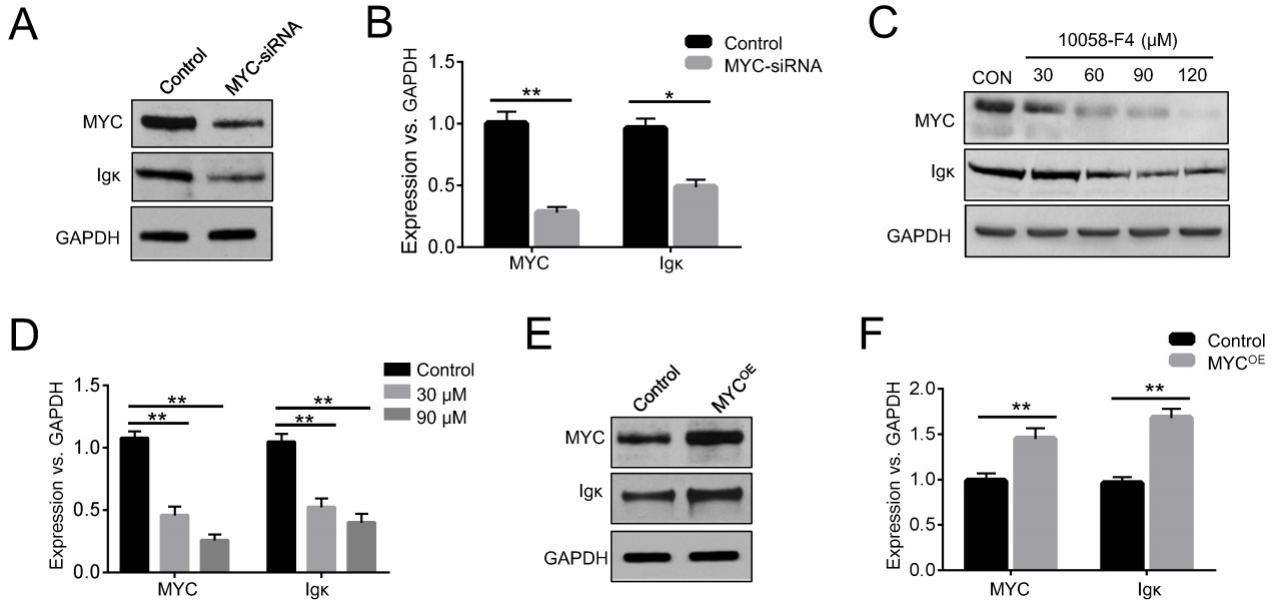
** MYC regulates Igκ expression in Raji cells.** (A) Western blot was performed to measure the levels of MYC and Igκ in Raji cells treated with MYC-siRNA or control-siRNA for 48 h. (B) Real-time PCR was used to measure MYC and Igκ transcription in control and MYC knockdown Raji cells. The levels of MYC and Igκ in Raji cells treated with 10058-F4 at different concentrations for 24 h were measured by western blot (C) and real-time PCR (D). Raji cells were transfected with control or MYC-expressing vectors (MYC^OE^) and incubated for 48 h, followed by western blot analysis (E) and real-time PCR (F) (*P<0.05, **P<0.01 compared to the control).

In BL cells, MYC is predominantly expressed from the translocated chromosome, whereas the normal allele is silent or expressed at a very low level 23. Raji cells have been widely used in the study of BL, as they harbor a typical translocation of MYC fused to the IgH gene 24. To investigate whether MYC has any effects on Ig expression, we knocked down MYC in Raji cells using siRNA and measured the levels of Igκ. The results indicated that interfering with MYC expression significantly suppressed Igκ expression, as shown by western blot and real-time PCR (Figure 1A, 1B). The reduction in Igκ mRNA levels upon MYC knockdown suggested that MYC regulates Igκ expression at the transcriptional level. Next, we treated the cells with 10058-F4, a MYC inhibitor, and found that the treatment reduced the levels of MYC protein (Figure 1C), which is in line with the results of previous studies 25, 26. As shown in Figure 1C-1E, MYC inhibition by 10058-F4 suppressed the expression of Igκ in a dose-dependent manner, whereas MYC overexpression (MYC^OE^) induced a higher level of Igκ expression compared to that of the control (CON). These data suggest that MYC acts as a positive regulator of Igκ expression in BL cells.

### Interfering with MYC and Igκ expression suppresses BL cell growth

**Figure 2:**
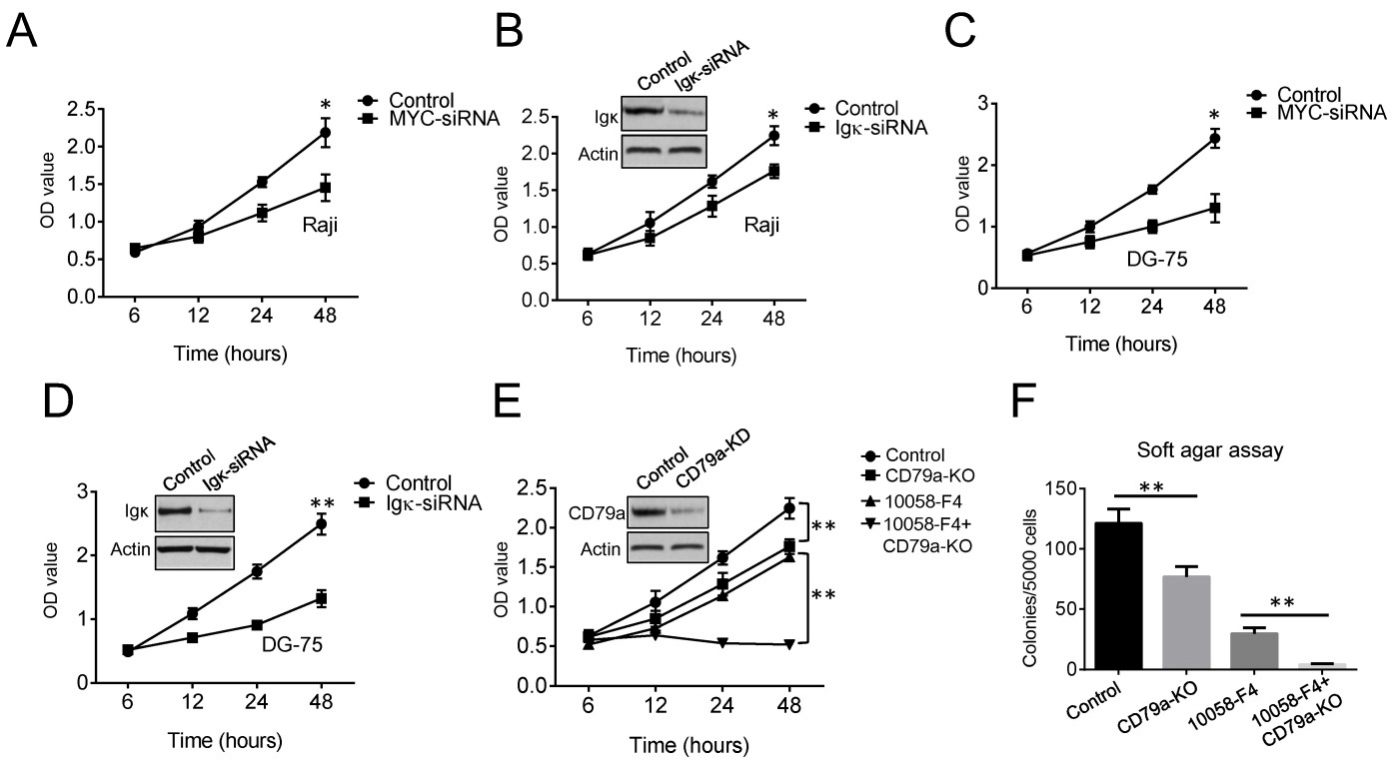
** Both MYC and Igκ are important for BL cell proliferation.** (A-D) The results of the cell proliferation assay show the growth of Raji and DG-75 cells with MYC or Igκ knockdown by siRNA. The effects of siRNA knockdown on Igκ expression were measured by western blot, and the results are shown in Figure 2B and Figure 2D. (E) The effect of Lenti-CRISPR knockout on the CD79a gene in Raji cells was measured by western blot. Then, a cell proliferation assay was performed to measure the growth of control or CD79a-KO cells with or without treatment with 30 μM 10058-F4. (F) Soft agar assays were used to detect colonies formed by control or CD79a-KO cells with or without treatment with 30 μM 10058-F4 (*P<0.05, **P<0.01 compared to the control).

MYC is a major oncogenic driver in BL. As expected, cell proliferation was markedly suppressed in MYC knockdown cells compared to that in control cells (Figure 2A). To test if BCR signaling is also required for the growth of BL cells, Igκ was knocked down using siRNA, and a cell proliferation assay was performed. The results indicated that the expression of Igκ is important for optimal cell growth (Figure 2B). Similar results were observed for another BL cell line, DG-75 (Figure 2C, 2D), which is in agreement with the results of previous studies showing that the survival of BL cells relies on the expression of both MYC and BCR 27, 28.

BCR signaling transduction is mediated by the CD79a/CD79b heterodimer (gα/Igβ). We next generated cell lines in which the CD79a gene was ablated with the CRISPR/Cas9 technique and found that depleting CD79a (CD79a-KO) suppressed tumor cell proliferation (Figure 2E). The wild-type and CD79a-KO cells were then treated with 30 μM 10058-F4, which only slightly altered the levels of MYC but not those of Igκ (Figure 1C). The treatment dramatically suppressed the growth of CD79a-KO cells compared to that of the wild-type cells (Figure 2E). To further test whether the targeting of MYC or/and BCR signaling in Raji cells can impair anchorage-independent growth, which is a hallmark of tumorigenesis, a soft agar assay was performed. The results indicated that either 10058-F4 treatment or CD79a depletion significantly suppressed the formation of colonies by Raji cells in soft agar, and maximal suppression was achieved in CD79a-KO cells that were treated with 10058-F4, suggesting that the combined targeting of MYC and BCR signaling may produce more potent anti-tumoral efficacy than either of the individual treatments alone.

### MYC promotes Igκ expression by recruiting AP1 to enhancers

**Figure 3:**
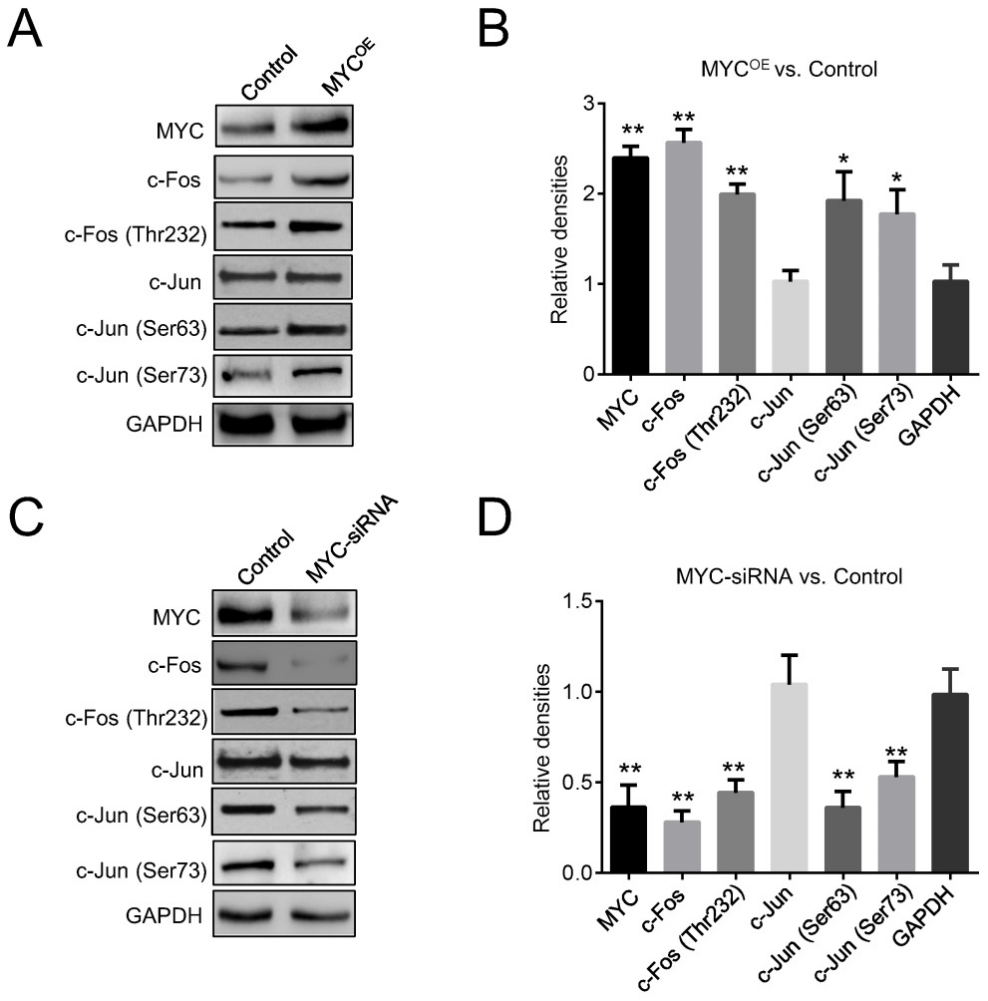
** MYC regulates AP1 activity.** (A) Western blot was performed for control and MYC-overexpressing Raji cells (MYC^OE^) to determine the levels of c-Fos, c-Jun, p-c-Jun at Ser 63 and Ser 73, and p-c-Fos at Thr 232. (B) The quantitated densities (MYC^OE^ vs. Control) of the western blot bands in Figure 3A. (C) Western blot shows the amounts of c-Jun, c-Fos, p-c-Fos, and p-c-Jun in control and MYC knockdown Raji cells. (D) The quantitated densities (MYC-siRNA vs. Control) of the western blot bands in Figure 3C (*p<0.05, **p<0.01 relative to the GAPDH).

Two enhancers, Ei and E3ʹ, are essential for the expression of the Igκ gene in regular B-cells, but whether they are required for MYC-induced Igκ upregulation in BL cells is not clear. AP1 binding sites have been identified in both Ei and E3ʹ as well as in the IgH 3ʹ enhancer, and they function as positive elements that induce enhancer activity 10, 29, 30. We first examined the effects of MYC on the expression of the two main AP1 subunits, c-Jun and c-Fos. As shown in Figure 3A and 3B, c-Fos was upregulated in MYC^OE^ Raji cells, whereas the expression of c-Jun remained unchanged. Consistently, Raji cells with MYC knockdown exhibited a reduced level of c-Fos compared to that of control cells (Figure 3C, 3D). Phosphorylation by c-Jun (Ser 63 and Ser 73) and c-Fos (Thr 232) are crucial for the AP1 heterodimer to bind DNA and to activate target genes 31. As shown in Figure 3A-3D, ectopic expression of MYC promoted c-Jun and c-Fos phosphorylation at these sites, whereas knocking down MYC diminished the levels of phosphorylation, implying that MYC may increase AP1 activity by inducing c-Jun and c-Fos phosphorylation and thus enhance Igκ expression.

**Figure 4:**
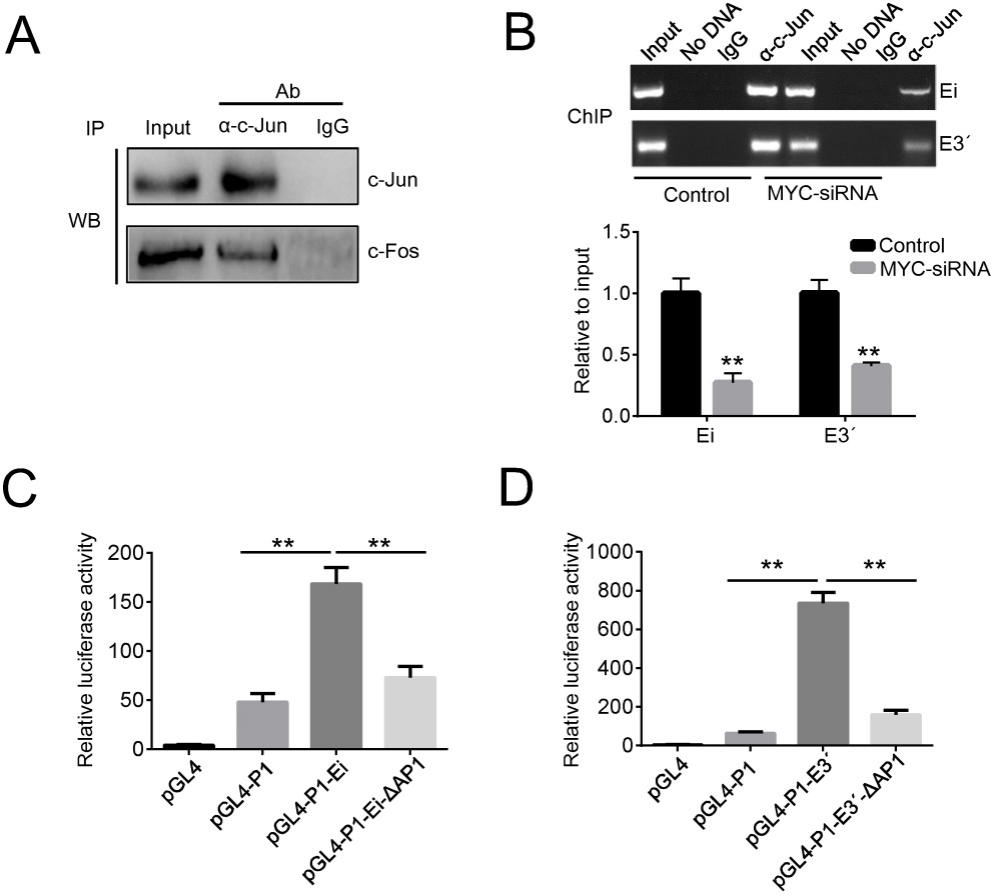
** MYC promotes the recruitment of AP1 to the enhancers.** (A) The protein-protein interaction of c-Jun and c-Fos in Raji cells was demonstrated by a co-IP assay. (B) ChIP assays were performed in control or MYC knockdown Raji cells. The impact of MYC knockdown on c-Jun binding to Ei and E3ʹ were determined by PCR (upper) and real-time PCR (bottom). (D) Raji cells were transfected with the reporter vectors indicated in the figure, and after 48 h of incubation, the enhancer activities were measured with luciferase reporter assays (*p<0.05, **p<0.01 compared to the control).

To further study the role of AP1 in Igκ regulation, we examined the protein-protein interaction between c-Jun and c-Fos with co-IP and then performed ChIP to detect the recruitment of c-Jun to the enhancers. As expected, the binding of c-Jun and c-Fos was readily detected by the co-IP assay in Raji cells (Figure 4A), while the ChIP results demonstrated that both Ei and E3ʹ were enriched in DNA precipitated by a c-Jun antibody (Figure 4B). These data suggest that c-Jun and c-Fos form AP1 heterodimers in BL cells and are recruited to Igκ enhancers. To directly test if AP1 binding is required for enhancer activity, a luciferase reporter assay was performed in Raji cells by introducing the following constructs, which contain either intact or AP1 binding site-deleted enhancers: pGL4-P1-Ei, pGL4-P1-E3ʹ, pGL4-P1-Ei-ΔAP1, and pGL4-P1-E3ʹ-ΔAP1. All constructs incorporated the P1 promoter, which is preferentially utilized to drive MYC overexpression in BL cells. The results demonstrated that the deletion of the AP1 binding sites impaired the activity of both Ei and E3ʹ (Figure 4C, 4D).

### MYC-induced Igκ upregulation is associated with JNK activation

**Figure 5:**
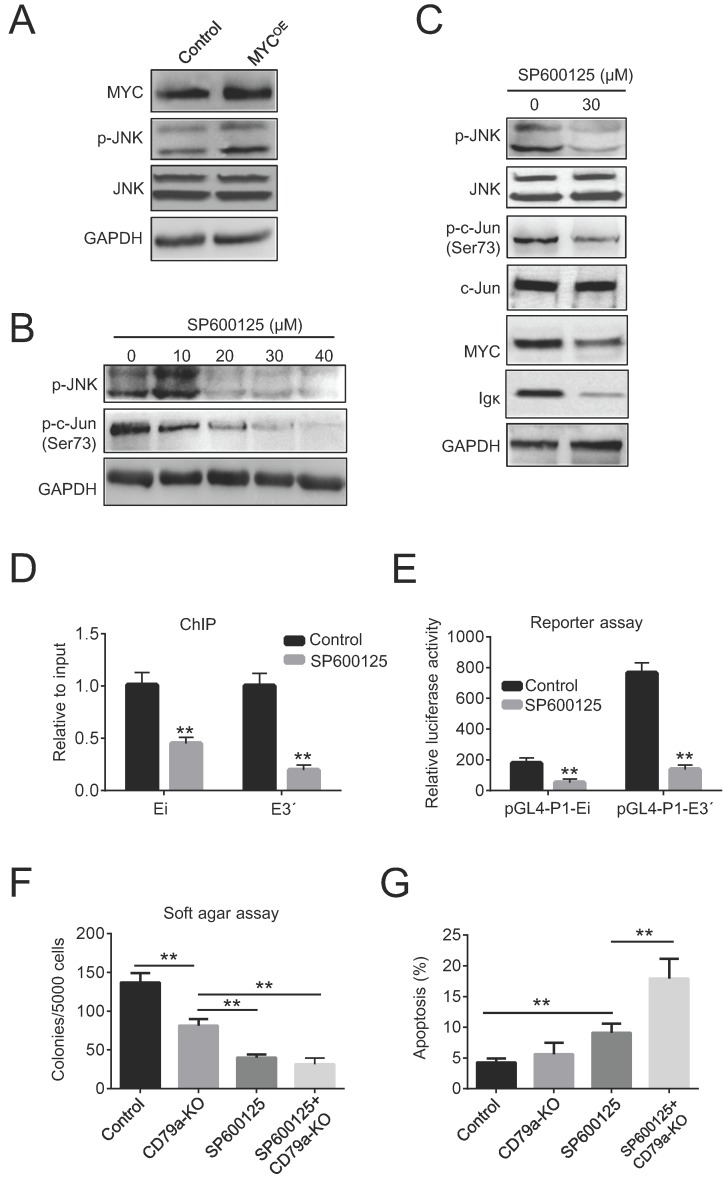
** Igκ upregulation is associated with JNK activation.** (A) The levels of p-JNK and JNK in control and MYC^OE^ cells were measured by western blot. (B) Raji cells were treated with SP600125 for 24 h at the indicated concentrations, and the levels of p-JNK and p-c-Jun were determined by western blot. (C) Western blot was used to measure the levels of p-c-Jun, c-Jun, p-JNK, JNK, MYC, and Igκ in Raji cells treated with or without SP600125 for 24 h. (D) Raji cells were incubated with SP600125 (30 μM) for 24 h to inhibit JNK activity, and its impact on the binding of c-Jun to Ei and E3ʹ was determined by a ChIP assay. (E) Raji cells were pretreated with SP600125 (30 μM) for 24 h and then transfected with the pGL4-P1-Ei or pGL4-P1-E3ʹ vector. The cells were cultured for 48 h in the presence of SP600125 (30 μM), and the enhancer activity was determined using a luciferase reporter assay and compared with that in control cells. (F) A soft agar assay was used to measure colony formation in wild-type and CD79a-KO Raji cells treated with or without 30 μM SP600125. (G) The levels of apoptosis in wild-type and CD79a-KO Raji cells treated with or without 30 μM SP600125 were determined according to the percentage of Annexin-V positive cells measured by using flow cytometry (*p<0.05, **p<0.01 compared to the control).

The role of c-Jun N-terminal kinase (JNK) as a positive regulator of c-Jun is well established 31. We reasoned that the increased phosphorylation of c-Jun could be caused by JNK activation in BL cells. Indeed, the ectopic expression of MYC in Raji cells increased JNK phosphorylation (Figure 5A). Next, Raji cells were treated with 10-40 µM of the JNK inhibitor SP600125 for 24 h. As expected, this reduced the levels of p-JNK in a dose-dependent manner (Figure 5B). We found that the levels of p-c-Jun, Igκ, and MYC were concurrently suppressed by SP600125, suggesting that JNK activity is associated with Igκ and MYC expression (Figure 5C). Moreover, SP600125 diminished the binding of c-Jun to the Igκ enhancers and suppressed the luciferase activity of pGL4-P1-Ei and pGL4-P1-E3ʹ (Figure 5D, 5E), indicating that the JNK/AP1 pathway is required for optimal Igκ expression. Finally, SP600125 exhibited potent anti-tumoral effects in Raji cells, especially in CD79a-KO cells, as indicated by the reduced colony formation in soft agar (Figure 5F). Notably, SP600125 increased apoptosis after 48 h of treatment in both control cells and, more significantly, CD79a-KO cells, as indicated by the percentage of Annexin-V positive cells (Figure 5G), suggesting that reduced colony formation caused by JNK inhibition is associated with increased apoptosis. Overall, these results suggest that MYC induces JNK activation and thus enhances the recruitment of AP1 to Ei and E3ʹ, leading to the upregulation of Igκ in BL cells.

## Discussion

AP1 is a transcription factor that controls many cellular processes, including differentiation, proliferation, apoptosis, and tumorigenesis 32. The structure of AP1 is a heterodimer composed of proteins belonging to the c-Fos, c-Jun, ATF, and JDP families 33. Studies using reporter constructs suggest that AP1 binds to the Ei and E3ʹ enhancers and promotes their activity 10, 30, 34. In agreement with this observation, we showed that the AP1 heterodimer c-Jun/c-Fos binds to the two enhancers of the endogenous Igκ gene, which is crucial for enhancer activity (Figure 4A, 4B). Notably, AP1 binding sites have also been identified in the 3ʹ IgH enhancer and are thought to play essential roles in IgH expression and lymphomagenesis 29, 35, 36. Further studies are needed to test whether AP1 is key in the synchronization of different Ig genes, by which an equal amount of heavy chains and light chains are expressed to efficiently generate immunoglobulin in B-cells.

As a transcriptional factor, AP1 activity is regulated by the levels of its subunit components as well as their posttranslational modifications, such as phosphorylation 32. We found that in BL cells, MYC induced the phosphorylation of both c-Fos and c-Jun by activating the JNK pathway, leading to increased AP1 binding and Igκ expression (Figure 3 and Figure 4B). Conversely, JNK inhibition diminished the recruitment of AP1 to the enhancers and reduced the levels of Igκ (Figure 5C-5E), suggesting that AP1 plays a pivotal role in MYC-mediated Igκ upregulation. On the other hand, oncogenic MYC expression in BL is actually driven by nearby Ig enhancers; hence, we reasoned that AP1 activity is required for the expression of the translocated MYC gene. Indeed, the levels of MYC and Igκ were concomitantly suppressed by JNK inhibition, which is associated with reduced AP1 binding and enhancer activity (Figure 5C-5E). We therefore propose that, by inducing JNK activation, MYC promotes the recruitment of AP1 to Ig enhancers, which in turn augments the expression of the MYC gene itself. It is plausible that during the early stages of tumorigenesis, the basal activity of the Ig enhancers leads to MYC gene expression, while the MYC/JNK/AP1 pathway amplifies the activity of the enhancers and thus sustains the high expression levels of MYC and BCR, both of which are directly associated with the malignant transformation of BL cells.

Burkitt's lymphoma (BL) is a highly proliferative B-cell neoplasm that is treated with intensive chemotherapy, which is often not tolerable for elderly patients because of its toxicity 4. Moreover, no effective targeted therapy has been approved for the treatment of BL to date 37. Gururajan et al. showed that JNK inhibitors show strong activity in suppressing MYC expression and BL cell proliferation 38. Similarly, Leventaki et al. reported that JNK activation is elevated in most cases of BL and that blocking the JNK pathway significantly inhibits tumor cell growth 39. However, the underlying mechanisms of the anticancer effects of JNK inhibition in BL are not clear. Our results imply that Ig enhancers might act as a platform on which AP1 plays a central role in the tumorigenesis of BL. While targeting MYC has been shown to be very challenging, we propose that disrupting MYC-mediated positive feedback through targeting JNK or AP1 may provide therapy for BL patients.

## Supplementary Material

Table S1: The oligonucleotide sequences of the primers and the sgRNAs (5′→3′). DOC S1: The generation of the constructs used for the luciferase reporter assay.

## Abbreviations

BL: Burkitt lymphoma
BCR: B-cell receptor
ChIP: Chromatin immunoprecipitation
Co-IP: Coimmunoprecipitation
E3′: 3′ enhancer
Ei: Intrinsic enhancer
IgH: Ig heavy chain
Igκ: Ig kappa
sgRNA: Single guide RNA
TFs: Transcription factors

## References

[1] Schmitz R, Ceribelli M, Pittaluga S, Wright G, Staudt LM (2014). Oncogenic mechanisms in Burkitt lymphoma. Cold Spring Harb Perspect Med.

[2] Mangani D, Roberti A, Rizzolio F, Giordano A (2013). Emerging molecular networks in Burkitt's lymphoma. J Cell Biochem.

[3] Dang CV (2012). MYC on the path to cancer. Cell.

[4] Molyneux EM, Rochford R, Griffin B (2012). Burkitt's lymphoma. Lancet.

[5] Hortnagel K, Mautner J, Strobl LJ (1995). The role of immunoglobulin kappa elements in c-myc activation. Oncogene.

[6] Polack A, Feederle R, Klobeck G, Hortnagel K (1993). Regulatory elements in the immunoglobulin kappa locus induce c-myc activation and the promoter shift in Burkitt's lymphoma cells. EMBO J.

[7] Wittekindt NE, Hortnagel K, Geltinger C, Polack A (2000). Activation of c-myc promoter P1 by immunoglobulin kappa gene enhancers in Burkitt lymphoma: functional characterization of the intron enhancer motifs kappaB, E box 1 and E box 2, and of the 3' enhancer motif PU. Nucleic Acids Res.

[8] Inlay MA, Tian H, Lin T, Xu Y (2004). Important roles for E protein binding sites within the immunoglobulin kappa chain intronic enhancer in activating Vkappa Jkappa rearrangement. J Exp Med.

[9] Malin S, McManus S, Cobaleda C (2010). Role of STAT5 in controlling cell survival and immunoglobulin gene recombination during pro-B cell development. Nat Immunol.

[10] Schanke JT, Marcuzzi A, Podzorski RP, Van Ness B (1994). An AP1 binding site upstream of the kappa immunoglobulin intron enhancer binds inducible factors and contributes to expression. Nucleic Acids Res.

[11] Zhou X, Xian W, Zhang J (2018). YY1 binds to the E3' enhancer and inhibits the expression of the immunoglobulin kappa gene via epigenetic modifications. Immunology.

[12] Yam-Puc JC, Zhang L, Zhang Y, Toellner KM (2018). Role of B-cell receptors for B-cell development and antigen-induced differentiation. F1000Res.

[13] Burger JA, Wiestner A (2018). Targeting B cell receptor signalling in cancer: preclinical and clinical advances. Nat Rev Cancer.

[14] Havranek O, Xu J, Kohrer S (2017). Tonic B-cell receptor signaling in diffuse large B-cell lymphoma. Blood.

[15] Young RM, Staudt LM (2013). Targeting pathological B cell receptor signalling in lymphoid malignancies. Nat Rev Drug Discov.

[16] Schmitz R, Young RM, Ceribelli M (2012). Burkitt lymphoma pathogenesis and therapeutic targets from structural and functional genomics. Nature.

[17] Refaeli Y, Young RM, Turner BC, Duda J, Field KA, Bishop JM (2008). The B cell antigen receptor and overexpression of MYC can cooperate in the genesis of B cell lymphomas. PLoS Biol.

[18] Moyo TK, Wilson CS, Moore DJ, Eischen CM (2017). Myc enhances B-cell receptor signaling in precancerous B cells and confers resistance to Btk inhibition. Oncogene.

[19] Varano G, Raffel S, Sormani M (2017). The B-cell receptor controls fitness of MYC-driven lymphoma cells via GSK3beta inhibition. Nature.

[20] Psathas JN, Doonan PJ, Raman P, Freedman BD, Minn AJ, Thomas-Tikhonenko A (2013). The Myc-miR-17-92 axis amplifies B-cell receptor signaling via inhibition of ITIM proteins: a novel lymphomagenic feed-forward loop. Blood.

[21] Sanjana NE, Shalem O, Zhang F (2014). Improved vectors and genome-wide libraries for CRISPR screening. Nat Methods.

[22] Zhou X, Updegraff BL, Guo Y (2017). PROTOCADHERIN 7 Acts through SET and PP2A to Potentiate MAPK Signaling by EGFR and KRAS during Lung Tumorigenesis. Cancer Res.

[23] Eick D, Bornkamm GW (1989). Expression of normal and translocated c-myc alleles in Burkitt's lymphoma cells: evidence for different regulation. EMBO J.

[24] Kiaei A, Onsori H, Alijani A, Andalib S, Ghorbian S, Sakhinia E (2016). Detection of t(8;14) c-myc/IgH gene rearrangement by long-distance polymerase chain reaction in patients with diffuse large B-cell lymphoma. Hematol Oncol Stem Cell Ther.

[25] Tarrado-Castellarnau M, de Atauri P, Tarrago-Celada J (2017). De novo MYC addiction as an adaptive response of cancer cells to CDK4/6 inhibition. Mol Syst Biol.

[26] God JM, Cameron C, Figueroa J (2015). Elevation of c-MYC disrupts HLA class II-mediated immune recognition of human B cell tumors. J Immunol.

[27] Corso J, Pan KT, Walter R (2016). Elucidation of tonic and activated B-cell receptor signaling in Burkitt's lymphoma provides insights into regulation of cell survival. Proc Natl Acad Sci U S A.

[28] Doose G, Haake A, Bernhart SH (2015). MINCR is a MYC-induced lncRNA able to modulate MYC's transcriptional network in Burkitt lymphoma cells. Proc Natl Acad Sci U S A.

[29] Grant PA, Thompson CB, Pettersson S (1995). IgM receptor-mediated transactivation of the IgH 3' enhancer couples a novel Elf-1-AP-1 protein complex to the developmental control of enhancer function. EMBO J.

[30] Liu H, Zheng H, Duan Z (2009). LMP1-augmented kappa intron enhancer activity contributes to upregulation expression of Ig kappa light chain via NF-kappaB and AP-1 pathways in nasopharyngeal carcinoma cells. Mol Cancer.

[31] Bubici C, Papa S (2014). JNK signalling in cancer: in need of new, smarter therapeutic targets. Br J Pharmacol.

[32] Gazon H, Barbeau B, Mesnard JM, Peloponese JM Jr (2017). Hijacking of the AP-1 Signaling Pathway during Development of ATL. Front Microbiol.

[33] Hess J, Angel P, Schorpp-Kistner M (2004). AP-1 subunits: quarrel and harmony among siblings. J Cell Sci.

[34] Pongubala JM, Atchison ML (1997). PU.1 can participate in an active enhancer complex without its transcriptional activation domain. Proc Natl Acad Sci U S A.

[35] Vincent-Fabert C, Fiancette R, Cogne M, Pinaud E, Denizot Y (2010). The IgH 3' regulatory region and its implication in lymphomagenesis. Eur J Immunol.

[36] Wang J, Boxer LM (2005). Regulatory elements in the immunoglobulin heavy chain gene 3'-enhancers induce c-myc deregulation and lymphomagenesis in murine B cells. J Biol Chem.

[37] Casulo C, Friedberg J (2015). Treating Burkitt Lymphoma in Adults. Curr Hematol Malig Rep.

[38] Gururajan M, Chui R, Karuppannan AK, Ke J, Jennings CD, Bondada S (2005). c-Jun N-terminal kinase (JNK) is required for survival and proliferation of B-lymphoma cells. Blood.

[39] Leventaki V, Drakos E, Karanikou M (2014). c-JUN N-terminal kinase (JNK) is activated and contributes to tumor cell proliferation in classical Hodgkin lymphoma. Hum Pathol.

