# Supplementary material for: JNK/AP1 Pathway Regulates MYC Expression and BCR Signaling through Ig Enhancers in Burkitt Lymphoma Cells
Source: J Cancer. 2020 Jan 1;11(3):610–8. doi: 10.7150/jca.34055 (PMC6959055; doi:10.7150/jca.34055)
Supplement: Supplementary file 1 — Table S1: The oligonucleotide sequences of the primers and the sgRNAs (5′→3′). DOC S1: The generation of the constructs used for the luciferase reporter assay. [file jcav11p0610s1.pdf]

SUPPLEMENTAL INFORMATION

Table S1: The oligonucleotide sequences of the primers and the sgRNAs (5′→3′). The sequences of the primers for realtime quantitative PCR (qPCR), and the sgRNAs for LentiCRISPR are listed below. The sgRNA sequences for the CD79a gene and the non-targeting control are from the Human CRISPR Knockout Pooled Library (GeCKO v2, references 21 in the main manuscript).

Genes and Assays	Directions	Sequences
Igκ qPCR	Forward	CCATCTGATGAGCAGTTGAAATCT
	Reverse	TCTGTGACACTCTCCTGGGAGTTA
MYC qPCR	Forward	CCTGGTGCTCCATGAGGAGAC
	Reverse	CAGACTCTGACCTTTTGCCAGG
GAPDH qPCR	Forward	GTCTCCTCTGACTTCAACAGCG
	Reverse	ACCACCCTGTTGCTGTAGCCAA
Ei for ChIP and qPCR	Forward	CTATCTGTTGACTTCTCCCAGCAA
	Reverse	AGGAAGTGGCTAGCTTCACTTCTG
E3′ for ChIP and qPCR	Forward	CACTCCACACCCTTTCAGAAGTT
	Reverse	GATATCAAACAAGGTTGGGGTTG
CD79a sgRNA for CRISPR		GAACCGAATCATCACAGCCG
Control sgRNA for CRISPR		CGCTTCCGCGGCCCGTTCAA

SUPPLEMENTAL INFORMATION

DOC S1: The generation of the constructs for the luciferase reporter assay. By modifying the pGL4-Basic vector from Promega (Madison, WI), we generated the four reporter constructs: pGL4-P1, pGL4-P1-E3′, pGL4-P1-Ei-ΔAP1, and pGL4-P1-E3′-ΔAP1, and the detailed methods are described as follows:

The MYC promoter P1 (186bp) was amplified from genomic DNA by PCR using the following primers (restriction enzyme sites are underscored): forward, CGGGGTACCATGCGAGGGTCTGGACGGCTGA; reverse, CCCAAGCTTAGCCGGTTTTCGGGGCTTTAT. The purified PCR products were inserted into the kpnI-HindIII sites in the pGL4 basic vector to generate pGL4-P1. The core region of the human Ei and the E3´were amplified from genomic DNA by PCR and cloned into the BamHI-SalI sites in the pGL4-P1vector to generate pGL4-P1-Ei and pGL4-P1-E3´, respectively. The sequences of the P1 promoter and the two enhancers are provided as follows: The primers used to amplify the Ei are as follows: forward, CGCGGATCCCTGACTTCTCCCTATCTGTTGAC; reverse, CCACGCGTCGACCCATTCTGAGGGCTTTGCATGCTTTTC. The following primers were used to amplify the E3´: forward, CGCGGATCCAACATGCCCCCAACCAGCCCCACC, reverse, CCACGCGTCGACCAGTGCATTTAGGTGAAGTGATATC.

Human MYC P1 promoter sequence (186bp):

ATGCGAGGGTCTGGACGGCTGAGGACCCCCGAGCTGTGCTGCTCGCGGCC

GCCACCGCCGGGCCCCGGCCGTCCCTGGCTCCCCTCCTGCCTCGAGAAGG

GCAGGGCTTCTCAGAGGCTTGGCGGGAAAAAGAACGGAGGGAGGGATCG

CGCTGAGTATAAAAGCCGGTTTTCGGGGCTTTATCTA

Human Ei sequence (575bp):

CTGACTTCTCCCTATCTGTTGACTTCTCCCAGCAAAAGATTCTTATTTTACAT

TTTAACTACTGCTCTCCCACCCAACGGGTGGAATCCCCCAGAGGGGGATTT

CCAAGAGGCCACCTGGCAGTTGCTGAGGGTCAGAAGTGAAGCTAGCCACT

TCCTCTTAGGCAGGTGGCCAAGATTACAGTTGACCTCTCCTGGTATGGCTG

AAAATTGCTGCATATGGTTACAGGCCTTGAGGCCTTTGGGAGGGCTTAGAG

AGTTGCTGGAACAGTCAGAAGGTGGAGGGGCTGACACCACCCAGGCGCA

GAGGCAGGGCTCAGGGCCTGCTCTGCAGGGAGGTTTTAGCCCAGCCCAGC

CAAAGTAACCCCCGGGAGCCTGTTATCCCAGCACAGTCCTGGAAGAGGCA

CAGGGGAAATAAAAGCGGACGGAGGCTTTCCTTGACTCAGCCGCTGCCTG

GTCTTCTTCAGACCTGTTCTGAATTCTAAACTCTGAGGGGGTCGGATGACG

TGGCCATTCTTTGCCTAAAGCATTGAGTTTACTGCAAGGTCAGAAAAGCAT

GCAAAGCCCTCAGAATGG

Human E3´ sequence (385bp):

AACATGCCCCCAACCAGCCCCACCTCAGACTGGTTATTACAGAGTTTCATG

GTTACTTGCCTGAGAAGATTAAAAAAAGTAATGCTACCTTATGAGGGAGAG

TCCCAGGGACCAAGATAGCAACTGTCATAGCAACCGTCACACTGCTTTGGT

CAAGGAGAAGACCCTTTGGGGAACTGAAAACAGAACCTTGAGCACATCTG

TTGCTTTCGCTCCCATCCTCCTCCAACAGGGCTGGGTGGAGCACTCCACAC CCTTTCAGAAGTTCCCAAGGCCCCGTGCACCTGGGGTCACAACAGGACCT GGCCAAGGCTGTGTCCAGCACTGGGATGGGAAGTAACACCAACCCCAACC TTGTTTGATATCACTTCACCTAAATGCACTG;

The AP1 binding sites: *TGACTCA* in the Ei and *AGCAACTGTCATAGCAACCGTCACA* in the E3´, have been identified in prior studies (References 19 and 30 in the main manuscript). We thus performed site-directed mutagenesis to delete the AP1 binding sites in the two enhancers, using a Q5 Site-Directed Mutagenesis Kit (NEB, Ipswich, MA). The primers used for deleting the AP1 site in Ei are as follows: forward, GCCGCTGCCTGGTCTTCTTCAGACCTGTTC; reverse, AGGAAAGCCTCCGTCCGCTTTTATTTCCCCTG. And the following primers were used to remove the AP1 binding site from E3´: forward, CTGCTTTGGTCAAGGAGAAGACCCTTTGGGGA; reverse, ATCTTGGTCCCTGGGACTCTCCCTCATAAGGT. The resulted constructs with AP1 binding site deleted are designated as pGL4-P1-Ei-ΔAP1 and pGL4-P1-E3´-ΔAP1, respectively. All the primers listed above are in 5'to 3' direction, and the constructs generated were verified by sequencing.
